# The role of type 1 angiotensin 2 receptor polymorphism in asthmatic patients

**DOI:** 10.1186/2045-7022-3-S1-P17

**Published:** 2013-05-03

**Authors:** Margarida Cortez, Andreia Matos, Joana Ferreira, Leonor Lopes, Angela Gil, Manuel Bicho

**Affiliations:** 1 CHLN-HSM, Portugal; 2 Lisbon Medical School, Genetic Department, Portugal

## Background

It is known that type 1 angiotensin II (Ang II) receptor (AGTR1) could be related with the pathogenesis of bronchial asthma. It is involved in Th polarization, through different signaling pathways modulating allergic airway inflammation, and also may participate in airway remodeling and bronchoconstriction, that could be related with AGTR1 polymorphism. The purpose of this study is to analyze the association between AGTR1 1166A/C (rs5186) gene polymorphism with asthma severity.

## Methods

Asthmatic patients : n=37; were compared with a control group of n=32 healthy blood donors. The AGTR1 1166A/C (rs5186) gene polymorphism was determined by PCR-RFLP (polymerase chain reaction- restriction fragment length polymorphism). Control of asthma assessed by validated instrument (ACQ7 and PAQLQ). Statistical analysis was performed with PASW version 18 establishing a significance level of p<0.05.

## Results

The mean age of the 37 asthmatics was 40 ± 18 years; minimum 7 and maximum 71; 24 females and 13 males; 35 Caucasians and 2 non-Caucasians; 34 atopics and 3 non-atopics; 22 with controlled and 15 with uncontrolled asthma. The control group for this polymorphism is in Hardy-Weinberg equilibrium (p>0.05). In asthmatics the frequencies of the allele A is 53% and the allele C is 47%; in controls: 64% and 36% respectively. There is no statistical difference between these groups (p>0.05). Genotypes in the asthmatics- AA: 29.7%; AC: 46%; CC: 24.3%; in control group- AA: 34.4%; AC: 59.4%; CC: 6.2%. There is no statistical difference between these groups (p>0.05). When we associate AGTR1 genotypes, there was a tendency, but not significant, between asthmatics and controls (CC vs AC+AA; p=0.086) being CC genotype more frequent in the asthma-group. In asthmatics, there is no statistical difference (p>0.05) in genotypes: between atopics and non atopics; controlled and uncontrolled asthma; males and females; by ethnic-group; and in the different age-groups.

## Conclusions

Our findings provided some evidence that there is a trend, although not significant, that AGTR1 gene A1166C polymorphism might be a genetic marker for the pathophysiology of allergic airway inflammation, remodeling and bronchoconstriction in asthmatic disease.

